# The SLC25A42 Transcript Is a Biomarker for Fetal Reprogramming in Response to Placental Insufficiency in Preterm Newborns Under 32 Weeks Gestation—A Pilot Study

**DOI:** 10.3389/fped.2020.00459

**Published:** 2020-08-28

**Authors:** Fu-Sheng Chou, Pei-Shan Wang

**Affiliations:** ^1^Department of Pediatrics, University of Kansas Medical Center, Kansas City, KS, United States; ^2^Division of Neonatology, Children's Mercy-Kansas City, Kansas City, MO, United States; ^3^Department of Pediatrics, Loma Linda University, Loma Linda, CA, United States; ^4^PXT Research & Data Analytics, LLC, Rancho Cucamonga, CA, United States

**Keywords:** placental insufficiency, transcriptional biomarkers, intrauterine growth restriction, transcriptome analysis, fetal reprogramming

## Abstract

**Introduction:** Timing of medical delivery of preterm newborns exposed to placental insufficiency is largely determined by umbilical artery blood flow and maternal clinical manifestations. There is a lack of tools to properly assess fetal body response to placental insufficiency before or upon delivery. Yet, short- and long-term comorbidities associated with placental insufficiency and the consequential intrauterine growth restriction may be a result of fetal response following prolonged stress. This study aims to establish a procedure to investigate fetal/neonatal transcriptional response to placental insufficiency as part of an initiative to identify cost-effective biomarkers for assessing fetal response to placental insufficiency.

**Methods:** A prospective pilot study involving newborns with birth gestation <32 weeks was conducted to compare gene expression profiles in whole blood collected at birth among three clinically distinct groups - preeclampsia without placental insufficiency (PE), placental insufficiency (PI), and non-PE/PI groups.

**Results:** Whole blood from 11, 3, and 6 newborns in the non-PE/PI, PE, and PI groups were obtained. A transcriptome analysis found that the majority of the genes were downregulated in the PI group, suggesting global transcriptional inactivation. Intriguingly, SLC25A42, which encodes a mitochondrial transporter for coenzyme A and adenosine-3′,5′-diphosphate, was significantly upregulated in the PI group.

**Conclusion:** Transcriptional biomarkers for assessing fetal response to placental insufficiency may provide a useful tool to better understand the pathophysiology of fetal reprogramming in response to placental insufficiency. The validity and the role of SLC25A42, as well as its correlation with short- and long-term neonatal outcomes, warrants further investigation.

## Introduction

Survival of extremely preterm newborns (EPNs, those that are born at 27 weeks gestation or less) and very preterm newborns (VPNs, those that are born between 28 and 31 weeks) have increased significantly in the past decade due to substantial improvement in clinical management and technology (1). Surviving E/VPNs are at increased risk for neurodevelopmental abnormalities, including cognitive impairment, learning disabilities, and mental health issues (2–4). Those E/VPNs who experienced intrauterine growth restriction (IUGR) are at even greater risk of adverse neurodevelopmental outcomes and cardiometabolic derangement (5, 6). IUGR has also been shown to cause a multitude of short- and long-term complications involving almost all organ systems (7).

The etiologies of IUGR can be categorized into maternal, fetal, and placental. Placental vasculopathy is considered one of the most common causes of IUGR. Placental vasculopathy causes maternal preeclampsia and eclampsia, and is associated with placental insufficiency, a condition where blood supply toward the growing fetus is compromised, resulting in chronic fetal hypoxia and nutrient deprivation. Prolonged placental insufficiency results in fetal reprogramming to ensure conservation of energy in the growing fetus. As a result, IUGR ensues. Fetal reprogramming may serve as the molecular basis of the short- and long-term complications of IUGR, yet the molecular and cellular mechanisms underlying fetal reprogramming remains largely unexplored. The current standard of practice for diagnosing placental insufficiency is based on detection of abnormal umbilical artery blood flow, including a pulsatility index higher than 95%, or the presence of absent or reverse end-diastolic flow (8). The diagnosis of IUGR by fetal weight estimation using non-invasive sonography is still a challenge, and the presence of IUGR may indicate that irreversible fetal reprogramming has already occurred (9).

Limitations in sonographic detection of placental insufficiency include the need for an experienced sonographer and relatively high cost; sonographic studies are also not always readily available to pregnant women who are at risk. More importantly, the tools for diagnosing placental insufficiency does not provide information with regard to fetal response to the intrauterine stress to guide clinical decision making in terms of delivery of the fetus vs. expectant management. Alternatively, transcriptional biomarkers for assessing global and/or targeted fetal gene regulation in response to placental insufficiency may be an ideal tool to determine whether fetal stress and reprogramming are present, and to what extent. In order to achieve this ultimate goal, we took an initiative and conducted a pilot study to establish a procedure for correlating fetal/neonatal whole blood transcriptome with placental insufficiency. We hypothesize that transcriptome profiles are altered in response to placental insufficiency-induced intrauterine stress in the growing fetuses.

## Methods

### Study Subjects

The study was approved by Institutional Review Board of the Human Research Protection Program at the University of Kansas Medical Center (KUMC) and the Office of Research Integrity at Children's Mercy-Kansas City. Pregnant women admitted to the Labor & Delivery unit at KUMC for threatened preterm delivery at <32 weeks gestation were screened for study participation. Informed consent for collecting neonatal blood upon delivery as well as maternal and neonatal clinical information was obtained after all questions were answered. Criteria for participation included delivery at <32 weeks gestation, singleton or dichorionic-diamniotic twin. Newborns were excluded if multiple congenital anomalies or congenital cytomegalovirus infection was diagnosed. Based on the absence or presence of maternal preeclampsia and placental insufficiency, E/VPNs were assigned to one of the three groups for comparison: [1] no maternal preeclampsia or placental insufficiency (the non-PE/PI group); [2] maternal preeclampsia but no placental insufficiency (the PE group); and [3] placental insufficiency (the PI group). Maternal preeclampsia diagnosis was based on maternal chart review, and placental insufficiency in this study was defined as abnormal UA blood flow, including a pulsatility index of >95%, absent end-diastolic flow, or reversed end-diastolic flow (8, 10). The grouping strategy was based solely on chart review of maternal diagnosis and the most recent umbilical Doppler findings prior to delivery without further considering underlying etiologies (e.g., TORCH infections, maternal smoking, pre-existing maternal hypertension, etc.). The indications for preterm delivery of E/VPNs in the non-PE/PI group were variable, mostly due to premature rupture of membrane or placental abruption.

### Blood Collection and Processing

One-half milliliter of whole blood was collected during umbilical catheter placement, typically within 1 h of birth. Blood was collected into a non-additive specimen tube and placed in a −20°C freezer within 30 min of collection. For RNA extraction, TRIzol™ (ThermoFisher Scientific, Waltham, MA, USA) was added directly to the frozen blood specimen following manufacturer's instructions. RNA concentration was measured using a Qubit fluorometer (ThermoFisher Scientific). For all samples, handling of RNA was performed by the same person in order to minimize variation introduced by sample processing. RNA was then stored in a −80°C freezer until use.

### RNA Sequencing and Transcriptome Analysis

RNA sequencing (paired-end 75 base pair sequences with a sequencing depth of 25 million reads per sample) was performed by using an Illumina NextSeq sequencer at the Oklahoma State University Genomics facility. RNA integrity was checked by using the Agilent 2200 TapeStation system (Agilent, Santa Clara, CA) prior to sequencing. Post-sequencing data processing, including demultiplexing, file format conversion, and quality trimming were also performed by the sequencing facility. Sequencing raw data has been deposited in GenBank (BioProject: PRJNA639860). Raw counts were downloaded in the fastq file format for quasi-mapping using *Salmon* (11). The Genome Reference Consortium Human Build 38 version was used as reference. The differential gene expression analysis was performed in R (version 3.6.1) and the RStudio (version 1.2) integrated development environment using the *DESeq2* package (12).

## Results

### Patient Characteristics

A total of 20 whole blood samples were collected, including 11 for the non-PE/PI group, 3 for the PE group, and 6 for the PI group. Patient characteristics are listed in the Table 1. No significant differences in the mean maternal age and the mean gestational age at birth were observed. As expected, the PI group had the lowest mean birth weight and mean birth weight z-score when compared to the non-PE/PI and the PE groups. The median 1 min APGAR score was lower in the PE and the PI groups, indicating higher likelihood of needing resuscitation in the delivery room among E/VPNs born to mothers with preeclampsia or placental insufficiency. However, median APGAR score at 5 min of life was comparable among all three groups.

**Table 1:** Maternal and neonatal characteristics.

**Group**	**No maternal preeclampsia/placental insufficiency (Non-PE/PI)**	**Maternal preeclampsia (PE)**	**Placental insufficiency (PI)**
Number	11	3	6
**MATERNAL CHARACTERISTICS**			
Maternal age (year)	29.5 ± 7.5	30.7 ± 2.9	29 ± 6.4
Non-Hispanic white (*n*)	10	3	4
Hispanic white (*n*)	0	0	1
Black (*n*)	1	0	1
**NEONATAL CHARACTERISTICS**			
Female (*n*)	9	2	2
Gestational age at birth (week)	27.5 ± 2.9	28.3 ± 3.2	27.4 ± 2.3
Birth weight (g)	1.125 ± 387	1.099 ± 325	836 ± 358
Birth weight z-score	0.3 ± 0.7	0 ± 0.6	−1.2 ± 0.7
Median APGAR at 1 min	7	3	5
Median APGAR at 5 min	8	7	8

### Transcriptome Analysis

**Figure 1:**
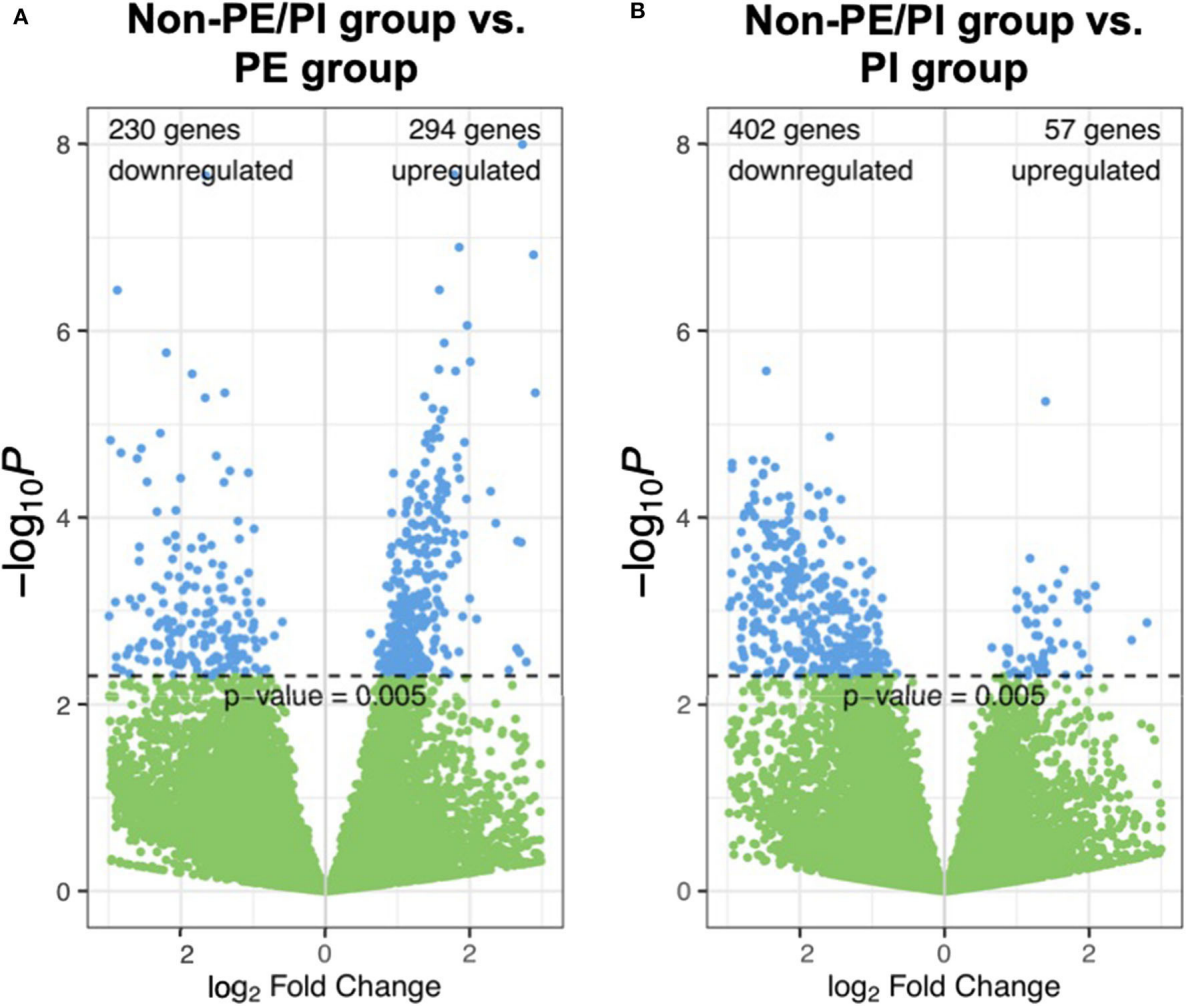
Volcano plots showing pairwise comparisons of gene expression between the non-maternal preeclampsia/placental insufficiency (non-PE/PI) and the PE groups **(A)**, as well as between the non-PE/PI and the PI groups **(B)**.

In volcano plots, we found that the majority of genes in the PI group were downregulated when compared to the non-PE/PI group; on the other hand, the numbers of the genes that were upregulated and downregulated were comparable between the non-PE/PI and the PE groups (Figure 1).

**Figure 2:**
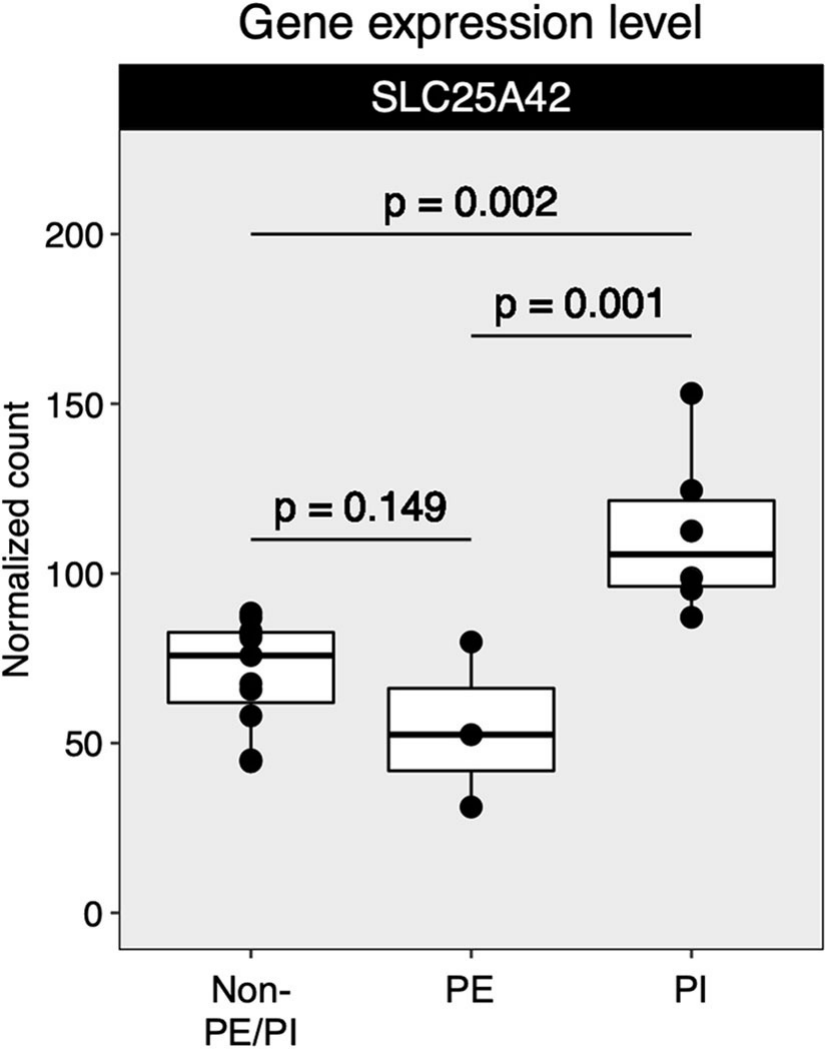
Comparison of SLC25A42 gene expression levels among non-maternal preeclampsia/placental insufficiency (non-PE/PI), maternal preeclampsia (PE), and the placental insufficiency (PI) groups.

Mapped sequences were called by *DESeq2* for differential gene expression (DGE) analysis. The *DESeq2* package applied a negative binomial statistical approach and utilized raw counts for DGE analysis (12). To identify candidate biomarkers for placental insufficiency, we searched for genes that were not differentially expressed between the non-PE/PI and the PE groups but were differentially expressed between the non-PE/PI and the PI groups. We found that SLC25A42, a gene that encodes a mitochondrial transporter for Coenzyme A and adenosine-3′,5′-diphosphate, was significantly upregulated in the PI group when compared to the other two groups (Figure 2).

## Discussion

Placental insufficiency is a common pregnancy complication which may lead to IUGR as well as multi-system short- and long-term adverse outcomes in the offspring. This report detailed findings from a pilot study aiming to establish a process for identifying transcriptional biomarkers for use in assessing fetal response to placental insufficiency. The long-term goal is to develop useful biomarkers to better examine fetal stress and reprogramming in response to placental insufficiency at its early stage before IUGR becomes detectable by conventional imaging studies, and to assist clinicians in determining the most optimal timing for delivery in order to prevent long-term comorbidities from IUGR.

In this pilot study, we showed that the process of blood collection from E/VPNs for RNA extraction, sequencing, and the subsequent transcriptome analysis for candidate transcriptional biomarker identification is feasible. We chose to collect circulating blood from the E/VPNs rather than from the umbilical cord due to logistic reasons (i.e., lack of easy access to placenta and umbilical cords after delivery) as well as the routine practice of delayed cord clamping during the delivery process in this population, which has been shown to improve short- and long-term outcomes (13). Moreover, blood collected upon umbilical catheter placement prior to fluid infusion were thought to contain cell types that are adequate for assessing fetal response to the intrauterine stress. As there was no additional physical stress associated with blood collection, we experienced no procedure-related complications in our small cohort. Although interventions provided to the newborns during delivery room resuscitation may potentially cause changes in gene expression, it was unlikely to occur, as we were able to collect and freeze whole blood within the first 1–2 h of life.

Several studies have successfully identified candidate biomarkers (metabolites or epigenetic alterations) from bodily fluids during pregnancy or upon delivery for correlation with pregnancy complications and the well-being of the offspring (14, 15). Transcriptome profiling with the use of RNA sequencing technology has emerged as a mainstream methodology in studying biological activities during normal development and functioning, as well as molecular responses to disturbances in biological homeostasis. It provides insights into how organisms react and adapt to changes in the environment at the cellular and molecular levels, and the signaling pathways involved (16). A recent landmark paper by Ngo et al. described the use of transcriptome profiles of maternal cell-free RNA derived from fetal tissues in combination with machine learning algorithms to identify candidate transcriptional biomarkers for preterm birth (17). In a similar manner, candidate transcriptional biomarkers short-listed from the current study or from future endeavor with a larger cohort may be further validated in fetal tissue-derived cell-free RNA in maternal blood prior to birth to quantify fetal response at the first sign of placental insufficiency, or even as a routine screening test in the high-risk population. Additionally, with its simplicity, serial blood testing may be performed on the at-risk pregnant women to closely trend fetal response to placental insufficiency.

Little is known about the role of SLC25A42 in response to the chronic hypoxia and nutrient deprivation associated with placental insufficiency. As the name implies, SLC25A42 is a member of the solute carrier family which serves as a transporter for Coenzyme A and adenosine 3′,5′-diphosphate in human mitochondria, suggesting that it plays a role in energy production and utilization (18). The significance of its upregulation in the context of placental insufficiency remains elusive and may warrant further molecular studies. Additionally, it would be interesting to correlate SLC25A42 expression levels with long-term outcomes of neonates in future studies. It is essential to emphasize that, because of the small cohort size in this feasibility study, the role of SLC25A42 as a potential transcriptional biomarker for placental insufficiency requires further confirmation with a larger study.

The timing of delivery in pregnancies complicated by preeclampsia or placental insufficiency is controversial, especially when the clinical issue develops at a gestational age that does not imply favorable neonatal outcomes. The detection of IUGR may play a role in clinical decision making, as it indicates ongoing and prolonged intrauterine stress. However, in such case, fetal reprogramming likely has already occurred, and therefore the associated short-term and long-term adverse outcomes (e.g., neurodevelopmental abnormalities and cardiometabolic risks) may have already become irreversible. Indeed, a recent study suggested that exposure to placental insufficiency by itself was sufficient to significantly alter postnatal growth trajectories, likely due to fetal reprogramming (10). Therefore, it is very likely that the developmental trajectory of the fetus is already altered and compromised when IUGR becomes detectable clinically. On the other hand, detection of placental insufficiency by Doppler studies without adequate assessment of fetal response may potentially lead to unnecessary premature delivery of fetuses that are not compromised. When cost-effect biomarkers for assessing fetal response to placental insufficiency become available for clinical use, it may be reasonable to take fetal response into consideration when determining the best timing for medical delivery of fetuses exposed to placental insufficiency.

Limitations of the study included a small sample size in each group and the heterogeneous nature of the non-PE/PI group without proper statistical adjustment due to small sample size. It also needs to be emphasized that the non-PE/PI group, although treated as the reference group for comparison in this pilot study, is not a conventional “control” group, as all preterm deliveries occur for a reason, which may have altered transcriptional regulations in different ways. The study was based on an assumption that a subset of transcripts was uniquely regulated in response to PE and/or PI. The study also did not take gender differences into consideration. Gender disparities in fetal response to IUGR has been well-established and must be addressed in future large-scale studies (19). Blood collected during umbilical catheter placement, albeit simple, contains a heterogenous population of nucleated cells and may confound biomarker identification. The most optimal site for blood sampling for RNA extraction is related to clinical questions to be solved, and blood samples collected from various sites may be further compared in future studies. From the sequencing standpoint, sequencing depth in this study may not be sufficiently robust to detect transcripts expressed at very low copy numbers. Overall, precautions must be taken when interpreting findings from this pilot study.

In conclusion, fetal transcriptional biomarkers may be a useful tool for assessing fetal response to placental insufficiency. Analysis of transcriptional alterations may also provide insight into the molecular basis of fetal reprogramming and the signaling pathways involved. A larger prospective study with clinical correlation, especially the long-term well-beings of the V/EPNs, is needed.

## Data Availability Statement

Sequencing raw data has been deposited in GenBank (BioProject: PRJNA639860).

## Ethics Statement

The studies involving human participants were reviewed and approved by Institutional Review Board of the Human Research Protection Program at the University of Kansas Medical Center and the Office of Research Integrity at Children's Mercy-Kansas City. Written informed consent to participate in this study was provided by the participants' legal guardian/next of kin.

## Author Contributions

F-SC conceptualized the study, collected whole blood from very preterm newborns, analyzed the RNA sequencing data, and prepared the manuscript. P-SW performed RNA extraction for RNA sequencing, analyzed the RNA sequencing data, and prepared the manuscript. All authors contributed to the article and approved the submitted version.

## Conflict of Interest

P-SW became the owner of PXT Research & Data Analytics, LLC after completion of the research work. The remaining author declares that the research was conducted in the absence of any commercial or financial relationships that could be construed as a potential conflict of interest.

## References

[1] Stoll BJ Hansen NI Bell EF Walsh MC Carlo WA Shankaran S. Trends in care practices, morbidity, and mortality of extremely preterm neonates, 1993-2012. JAMA. (2015) 314:1039–51. 10.1001/jama.2015.10244 26348753 PMC4787615

[2] Botellero VL Skranes J Bjuland KJ Løhaugen GC Håberg AK Lydersen S. Mental health and cerebellar volume during adolescence in very-low-birth-weight infants: a longitudinal study. Child Adolesc Psychiatry Ment Health. (2016) 10:6. 10.1186/s13034-016-0093-8 26985236 PMC4793750

[3] Costa DS Miranda DM Burnett AC Doyle LW Cheong JLY Anderson PJ. Executive function and academic outcomes in children who were extremely preterm. Pediatrics. (2017) 140:1–10. 10.1542/peds.2017-0257 28853418

[4] Botellero VL Skranes J Bjuland KJ Håberg AK Lydersen S Brubakk A-M. A longitudinal study of associations between psychiatric symptoms and disorders and cerebral gray matter volumes in adolescents born very preterm. BMC Pediatr. (2017) 17:45. 10.1186/s12887-017-0793-0 28143492 PMC5286868

[5] Korzeniewski SJ Allred EN Joseph RM Heeren T Kuban KCK O'Shea TM. Neurodevelopment at age 10 years of children born <28 weeks with fetal growth restriction. Pediatrics. (2017) 140:1–10. 10.1542/peds.2017-0697 29030525 PMC5654396

[6] Ramírez-Vélez R Correa-Bautista JE Villa-González E Martínez-Torres J Hackney AC García-Hermoso A. Effects of preterm birth and fetal growth retardation on life-course cardiovascular risk factors among schoolchildren from Colombia: the FUPRECOL study. Early Hum Dev. (2017) 106–7:53–58. 10.1016/j.earlhumdev.2017.02.001 28193574

[7] Sharma D Shastri S Sharma P. Intrauterine growth restriction: antenatal and postnatal aspects. Clin Med Insights Pediatr. (2016) 10:67–83. 10.4137/CMPed.S40070 27441006 PMC4946587

[8] Unterscheider J Daly S Geary MP Kennelly MM McAuliffe FM O'Donoghue K. Optimizing the definition of intrauterine growth restriction: the multicenter prospective PORTO Study. Am J Obstet Gynecol. (2013) 208:290.e1–6. 10.1016/j.ajog.2013.02.007 23531326

[9] Bardien N Whitehead CL Tong S Ugoni A McDonald S Walker SP. Placental insufficiency in fetuses that slow in growth but are born appropriate for gestational age: a prospective longitudinal study. PLoS ONE. (2016) 11:e0142788. 10.1371/journal.pone.0142788 26730589 PMC4701438

[10] Chou F-S Yeh H-W Chen C-Y Lee GT Parrish MR Omede M. Exposure to placental insufficiency alters postnatal growth trajectory in extremely low birth weight infants. J Dev Orig Health Dis. (2019) 4:1–8. 10.1017/S2040174419000564 31581967

[11] Patro R Duggal G Love MI Irizarry RA Kingsford C. Salmon provides fast and bias-aware quantification of transcript expression. Nat Methods. (2017) 14:417–9. 10.1038/nmeth.4197 28263959 PMC5600148

[12] Love MI Huber W Anders S. Moderated estimation of fold change and dispersion for RNA-seq data with DESeq2. Genome Biol. (2014) 15:550. 10.1186/s13059-014-0550-8 25516281 PMC4302049

[13] Kresch MJ. Management of the third stage of labor: how delayed umbilical cord clamping can affect neonatal outcome. Am J Perinatol. (2017) 34:1375–81. 10.1055/s-0037-1603733 28591905

[14] Maitre L Fthenou E Athersuch T Coen M Toledano MB Holmes E. Urinary metabolic profiles in early pregnancy are associated with preterm birth and fetal growth restriction in the Rhea mother–child cohort study. BMC Med. (2014) 12:110. 10.1186/1741-7015-12-110 25012562 PMC4094172

[15] Hodyl NA Roberts CT Bianco-Miotto T. Cord blood DNA methylation biomarkers for predicting neurodevelopmental outcomes. Genes. (2016) 7:117. 10.3390/genes7120117 27918480 PMC5192493

[16] Jiang Z Zhou X Li R Michal JJ Zhang S Dodson MV. Whole transcriptome analysis with sequencing: methods, challenges and potential solutions. Cell Mol Life Sci. (2015) 72:3425–39. 10.1007/s00018-015-1934-y 26018601 PMC6233721

[17] Ngo TTM Moufarrej MN Rasmussen M-LH Camunas-Soler J Pan W Okamoto J. Noninvasive blood tests for fetal development predict gestational age and preterm delivery. Science. (2018) 360:1133–6. 10.1126/science.aar3819 29880692 PMC7734383

[18] Fiermonte G Paradies E Todisco S Marobbio CMT Palmieri F. A novel member of solute carrier family 25 (SLC25A42) is a transporter of Coenzyme A adenosine 3′,5′-diphosphate in human mitochondria. J Biol Chem. (2009) 284:18152–9. 10.1074/jbc.M109.014118 19429682 PMC2709381

[19] Alur P. Sex differences in nutrition, growth, and metabolism in preterm infants. Front Pediatr. (2019) 7:22. 10.3389/fped.2019.00022 30792973 PMC6374621

